# Delayed effects of radiation exposure in a C57L/J mouse model of partial body irradiation with ~2.5% bone marrow shielding

**DOI:** 10.3389/fpubh.2024.1349552

**Published:** 2024-03-12

**Authors:** Tyler Beach, James Bakke, J. Tyson McDonald, Edward Riccio, Harold S. Javitz, Denise Nishita, Shweta Kapur, Deborah I. Bunin, Polly Y. Chang

**Affiliations:** SRI International, Menlo Park, CA, United States

**Keywords:** C57L/J mouse, partial body irradiation, animal models, ARS, DEARE, medical countermeasures against radiation

## Abstract

**Introduction:**

Mouse models of radiation injury are critical to the development of medical countermeasures (MCMs) against radiation. Now that MCMs against hematopoietic acute radiation syndrome (H-ARS) have achieved regulatory approval, attention is shifting to develop MCMs against the adverse effects of gastrointestinal acute radiation syndrome (GI-ARS) and delayed effects of acute radiation exposure (DEARE). The C57L/J mouse model of partial body irradiation (PBI) with 2.5% bone marrow shielding (BM2.5) is being leveraged to examine both GI-ARS and DEARE effects. Within days of PBI, mice may develop H- and GI-ARS followed several months later by DEARE as a multi-organ injury, which typically involves the lung and kidney (L- and K-DEARE, respectively). The objective of this manuscript is to describe the dose response relationship and progression of radiation injury in the C57L/J mouse and to evaluate its suitability for use in DEARE MCM testing.

**Materials and methods:**

In two separate studies conducted over 2 years, male and female C57L/J mice were exposed to PBI BM2.5 with one hindlimb shielded from radiation, representing ~2.5% bone marrow shielding/sparing. Mice were X-ray irradiated at doses ranging from 9 to 13 Gy at 10 to 12 weeks of age for the purposes of assessing ARS survival at 30 days and DEARE survival at 182 days post-irradiation. Clinical indicators of ARS and DEARE were determined by clinical observations, body weights, hematology, clinical chemistry, magnetic resonance imaging (MRI) of lung, and histopathology of selected tissues.

**Results:**

C57L/J mice developed canonical ARS responses of hematopoietic atrophy and gastrointestinal injury resulting in dose dependent mortality at doses ≥11 Gy between 1- and 15-days post-irradiation. In animals that survived ARS, DEARE associated mortality occurred in dose dependent fashion at ≥9 Gy for both sexes between 60- and 159-days post-irradiation with histopathology examinations indicating lung injury as the primary cause of death in moribund animals.

**Conclusion:**

The PBI BM2.5 C57L/J mouse model reliably produced known H- and GI-ARS effects at doses greater than those resulting in DEARE effects. Because of this, the C57L/J mouse can be used to test MCMs against L-DEARE injury, while avoiding ARS associated mortality.

## Introduction

Following a radiological disaster, the effects of radiation exposure are complex and may be long lived. Exposed individuals can experience an acute radiation syndrome (ARS) in the short term, i.e., days and weeks after exposure, and later go on to develop delayed effects (DEARE) in the weeks and months that follow. This multi-organ injury (1) impacts hematopoietic and gastrointestinal tissues acutely (H- and GI-ARS), and survivors of those injuries may later develop lung and kidney delayed effects of acute radiation exposures (L-DEARE and K-DEARE) (2). Numerous pathological processes including inflammation, fibrosis, chronic oxidative stress, senescence, genomic damage, endothelial death, and coagulopathy contribute to this highly complex multi-organ injury (1, 2).

Preclinical development of medical countermeasures (MCMs) against radiation injury is highly reliant on the development of animal models which mirror the clinical course of injury in humans. Such studies must be conducted under the U.S. Food and Drug Administration (FDA) Animal Rule (21 CFR 314.600-650 for drugs; 21 CFR 601.90-95 for biologics) which requires that when human efficacy studies would be unethical to perform, safety and efficacy shall be validated in at least two animal species or a single species that has been sufficiently well-characterized (3). Models used in MCM efficacy testing should be well-described with respect to the natural history of ARS or DEARE and be supported by institutional lethality studies to determine the dose response relationship (DRR) and probit estimates for lethality. This allows for a greater understanding of the impacts of radiation on various tissues and organ systems, can provide a basis for the determination of whether a MCM mitigates such damages, and can reveal a mechanism of action for the reduction of ARS or DEARE associated tissue injury and mortality (4).

Rodent (mouse and rat) models are ideal for countermeasures testing because certain species and strains re-capitulate many of the clinical aspects of radiation exposure and because the relatively low cost of rodent studies allows for multiple investigations, testing of both males and females, and sufficiently powered statistical analyses. For example, the total body irradiation (TBI) H-ARS C57BL6/J mouse model was used as the small animal model instrumental in demonstrating efficacy of H-ARS countermeasures (5–7). However, the TBI/H-ARS model is a poor choice for investigating DEARE because the TBI/H-ARS model uses radiation doses that are lower than those required for GI-ARS or DEARE injury (8). For the development of delayed effects, models of partial body irradiation (PBI) with bone marrow shielding are becoming more prevalent because they allow investigators examining the sequalae of radiation syndromes from H-and GI-ARS to L-DEARE and/or K-DEARE, much like would be experienced by victims of a radiological disaster (9).

The objective of this manuscript is to describe the natural history of radiation injury in C57L/J mice and generate probit estimates for lethality after PBI. The C57L/J strain is of interest because it has been shown to reliably develop L-DEARE following PBI with bone marrow sparing (10), TBI + whole thorax lung irradiation (WTLI) (11), and in the WTLI only (12) model, using exposures that do not result in a high degree of ARS associated mortality. Furthermore, earlier studies have shown that the L/J strain reflects the pathological features of human radiation lung injury to a greater degree than mouse strains such as the C57BL6/J, C3H, CBA, C57BR/J, BALB/c, and A/J (12–14). Thus, C57L/J mice may be well-suited for testing MCMs against DEARE after the resolution of ARS.

To determine suitability of the PBI BM2.5 C57L/J mice for use as a DEARE model for the testing of MCMs, we evaluated 30 day ARS survival, and 180 day DEARE survival post-irradiation. In a second study, we examined clinically relevant indicators of DEARE at scheduled intervals. These included clinical observations, body weights, blood collections for hematology and clinical chemistry, and magnetic resonance (MR) imaging of the lungs. Clinical findings were supported with pathology examinations of macroscopic and microscopic tissues to describe the extent of organ injury. Samples were collected from moribund animals and at scheduled intervals up to 182 days post-irradiation during both the ARS and DEARE periods. The data presented here were gathered from two separate studies conducted over a period of 2 years, using mice that were irradiated at the same age, i.e., 10–12 weeks. The first study was a dose response study (Study 1), and mice were irradiated at a range of doses from 9 to 13 Gy to establish the 30- and 180- day probit estimates of lethality and Kaplan Meier survival plots of 30-day ARS and 180-day DEARE mortality. The second study (Study 2) was performed to gather clinical data showing the natural history and progression of DEARE using fewer doses (11–13 Gy) previously shown to result in DEARE associated morbidity.

We have previously published the results a similarly designed series of studies successfully describing the sequalae of ARS and DEARE in the PBI BM5 Wistar rat model (15). The studies described in this manuscript were performed at the same institution using the same equipment and following standard operating procedures (SOPs) and institutional practices. Animal handling, clinical endpoints, and analytical methods were similar to those used described in Beach et al. (15).

## Method

### Animals

Male and female C57L/J mice were obtained from Jackson Laboratories (Sacramento, CA and Bar Harbor, ME) at 10–12 weeks of age and generally housed three animals per cage. A large number of animals were required to conduct the two studies, with the breeding facility providing 12 separate shipments over a 6-month period. Twelve irradiation sessions were performed, staggered by shipment date. Housing, handling, acclimation, husbandry activities and irradiation were the same for all shipments and are guided by institutional standard operating procedures (SOPs).

Animals were acclimated for at least 3 days prior to experimentation and received Envigo Teklad global 18% Protein Rodent Diet 2018C. Water was provided *ad libitum*. Supportive care consisted only of hydrogel fluid supplement and water softened chow for up to 30 days after irradiation, or any time body weights dropped ≥15% of the pre-irradiation body weight, or ≥15% of the body weights benchmarked at 30 day intervals. No other supportive care or medical management were provided. Experiments were performed in an Association for Assessment and Accreditation of Laboratory Animal Care International (AAALAC) accredited facility, under a protocol that has been approved by the Institutional Animal Care and Use Committee (IACUC) at SRI.

### Irradiation

Mice were anesthetized (ketamine + xylazine) and restrained with the lower-hind leg shielded with 4 mm of lead to spare ~2.5% of the bone marrow. During irradiation on day 0, each mouse was confined in a separate plastic jig, with six mice irradiated simultaneously. Irradiation was performed in a customized Pantek HF320 X-ray cabinet irradiator (Branford, CT) at energy settings of 250 kVp and 5 mA, with 2 mm aluminum filtration, equivalent to a 0.4 mm Cu half value layer (HVL). The dose rate was ~1.0 Gy/min. To confirm hindlimb shielding effectiveness, Landauer nanodot™ (ND) thermoluminescent dosimeters (TLDs) were placed under the lead leg shield, directly on top of the hindlimb. In the highest dose group (13 Gy) the mean dose to the shielded leg was ≤ 0.3 Gy; lower mean doses to the shielded leg were reported for all other dose groups.

All exposures took place during the morning (i.e., 8 am−12:00 pm) to avoid circadian confounding conditions (16). Dosimetry was conducted prior to study start and confirmed immediately before and after each day of irradiation. Group sizes ranging from 24 to 126 were composed of the same number of males and females (Table 1). Study 1 animals received 0, 9, 10, 11, 12, and 13 Gy PBI. Study 2 animals received 0, 11, 12, or 13 Gy PBI under identical conditions to Study 1.

**Table 1:** Number of animals per treatment group per sex for Study 1 and 2 combined.

**Group**	**Irradiation dose (Gy)**	**No. of animals**	**No. of males**	**No. of females**
1	0	60	30	30
2	9	48	24	24
3	10	48	24	24
4	11	120	60	60
5	12	156	78	78
6	13	252	126	126
**Total number**		**684**	**342**	**342**

### Health monitoring

Clinical observations were reported every day for the first 30 days after irradiation, and twice weekly thereafter. Animals were checked daily for mortality. Clinical observations were recorded as qualitative measures, and rated slight, moderate, or extreme. Body weights were recorded before irradiation and three times weekly from days 1 to 90. After day 90, weights were reported once each week. Additional body weights were also recorded in accordance with pre-established body weight change health monitoring criteria.

### Magnetic resonance imaging (MRI)

A series of serial images for each animal in the MRI cohort were obtained at ~84, 112, 140, and 180-days post irradiation using a Bruker 7T PharmaScan instrument, a 40 mm body coil, and ParaVision 6.0.1 software (Bruker, Billerica, MA). Animals were anesthetized and maintained using isoflurane in 100% O_2_ through a nose cone. Breathing rate and body temperature were monitored continuously using a MR-compatible monitoring system and SAII TrendMap Viewer 6.02 (SA Instruments Inc., Stony Brook NY) program. Because MRI requires special handling, animals used for MRI were assigned to a separate cohort, and not included in the survival analysis. Initially, eight animals of each sex were assigned to the control group, and 12 of each sex each for the 11, 12, and 13 Gy groups. However, mortality substantially reduced the numbers available for imaging, particularly at the last two time points (Table 2).

**Table 2:** Number of animals for MRI per study day (sexes combined).

**Irradiation dose (Gy)**	**Study day**			
	**84**	**112**	**140**	**180**
0	16	16	15	12
11	15	20	3	4
12	12	11	0	0
13	15	13	0	0

Respiratory gating and fat suppression were applied during image acquisition. To analyze MR images, visually verified areas of increased signal intensity and tissue density, which may indicate inflammation, congestion, or fibrosis, were recorded. The area of 3 separate, manually delineated lung sections were captured around the mid-point of the thorax and recorded for each animal using Image J version 1.52s (2019), and areas of high signal intensity within each section were measured. At each timepoint, group means comparing the relative area of high signal intensity within the sections were compared with the age matched control group for each timepoint.

T2 TurboRARE scans were performed using the following settings: Rapid acquisition with relaxation enhancement (RARE) sequence with repetition time (TR) 851.92 ms, RARE factor 4, echo time (TE) of 15.74 ms, echo spacing 7.8 ms, 15–0.8 mm thick slices positioned coronally over the lungs, 50 × 25 mm field of view (FOV), and image matrix of 256 × 128.

### Hematology parameters

Blood samples were collected for hematology and clinical pathology analysis. Animals were first anesthetized with isoflurane and blood was collected from the retro orbital sinus. All bleeds were terminal. A minimum of 3 mice/sex/dose group were assigned to scheduled collection timepoints. Scheduled collections occurred when possible at 42, 84, 98, 133, and 182 days post-irradiation (Table 3). Samples were also collected from moribund animals whenever possible and pooled into either ARS (euthanized prior to day 30) and DEARE (euthanized after day 30) cohorts by dose groups. Standard hematology parameters were analyzed using the HESKA small volume analyzer (Antech Diagnostics Inc., Loveland CO), and clinical chemistry with the Cobas c-501 Chemistry Analyzer (Roche, Indianapolis, IN).

**Table 3:** Number of male/female mice with scheduled or ^*^unscheduled blood collections.

**Irradiation dose (Gy)**	**Study day**					ARS^*^	DEARE^*^
	**42**	**84**	**98**	**133**	**180**		
0	3/3	3/3	0/0	4/4	7/8		
11	5/5	5/5	3/3	0/6	2/1	0/0	6/5
12	5/5	4/2	4/6	1/1	0/0	1/4	17/12
13	5/5	0/2	8/5	3/0	0/0	4/3	25/14
**Total Numbers**	**36**	**24**	**29**	**19**	**18**	**12**	**79**

^*^Unscheduled collections from moribund animals between days 1–15 (ARS) or days 60–150 (DEARE).

### Histopathology

In Study 2, tissue samples were collected at scheduled intervals from a minimum of 3 mice/sex for non-irradiated controls (0 Gy), 11, 12, and 13 Gy irradiated animals at 42, 84, 98, 133, and 182 days post-irradiation (6, 12, 14, 19, and 26 weeks), and from moribund animals when possible. Tissues collected included: gross lesions (including tissue masses and abnormal lymph nodes), sternum (for marrow histology), colon, cecum, rectum, duodenum, ileum, jejunum, stomach, kidneys, liver, lungs with bronchi, pancreas, spleen, testes/ovaries, thymus, and urinary bladder. Lungs were formalin inflated at ~30 cm hydrostatic pressure for up to 10 min prior to embedding. Tissues were formalin fixed, paraffin embedded, cut to 5 μm sections, and stained with hematoxylin and eosin (H&E). Additional lung sections were stained for the identification of fibrotic lesions, using Masson's trichrome. Lesions were listed and coded by the specific topographic and morphologic diagnoses, distribution and severity by a board-certified veterinary pathologist. A four-step grading system (minimal, mild, moderate, and marked) defined gradable lesions for reporting and comparison. The pathologist was not blinded to the radiation status of the animals.

### Statistics

Body weight, clinical pathology, and Kaplan Meier survival figures were generated using GraphPad Prism 10.0.3 (GraphPad Software, San Diego, CA.). Clinical pathology and hematology results from Study 1 and 2 were pooled for analysis using the rank sum test with *post-hoc* Benjamini–Hochberg correction for multiple comparisons. A log-rank test for survival function for each radiation dose as compared with controls, and probit estimates of radiation doses for lethality at 30 and 180 days were generated using Stata SE v14.2 for Windows (StataCorp LLC, College Station, TX).

## Results

### Survival

**Figure 1:**
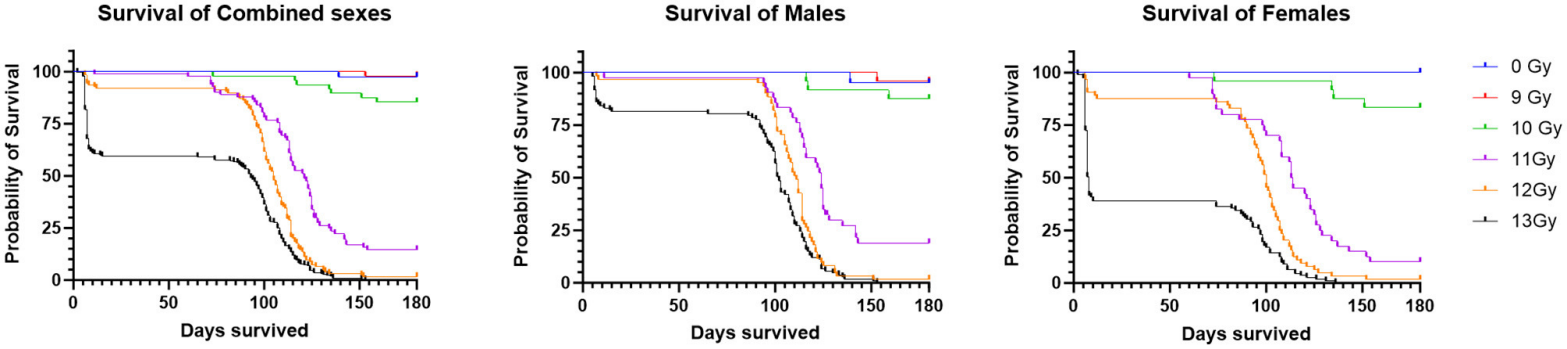
Kaplan Meier survival of PBI BM2.5 C57L/J mice for the combined sexes, males and females from Study 1 and 2 combined. For survival comparisons, the number of animals (N) for each group and sex were as follows; 0 Gy, 40– 20 M/20 F, 9 Gy, 48– 24 M/24 F, 10 Gy, 58– 29 M/29 F, 11 Gy, 84– 42 M/42 F, 12 Gy, 124– 62 M/62 F, and 13 Gy, 222– 111 M/111 F.

Two important findings are seen in the Kaplan Meier survival plots for the combined result of Study 1 and 2 for the sexes combined, males alone, or females alone (Figure 1). First is a dose dependent decrease in survival, and secondly there are two distinct periods of mortality due to either ARS (days 1–15) or DEARE (days 60–159). Survival percentages at 30 days following ARS mortality, and 180 days following DEARE associated mortality are shown in Table 4 for combined sexes, males alone, and females alone.

**Table 4:** Survival results at day 30 (studies 1 and 2 combined) and day 180 (Study 1).

**Group**	**Radiation dose (Gy)**	**30 day survival**			**180 day survival**		
		**Males**	**Females**	**Both sexes**	**Males**	**Females**	**Both sexes**
1	0	100%	100%	100%	100%	100%	98%
2	9	100%	100%	100%	95%	100%	98%
3	10	100%	100%	100%	86%	83%	85%
4	11	96%	100%	98%	19%	10%	14%
5	12	96%	87%	91%	2%	2%	2%
6	13	78%	39%	60%	0%	0%	0%

There was no mortality among non-irradiated controls, except for a single male that was found dead on day 139, with no prior clinical findings or indications of poor health.

H- and GI-ARS associated deaths occurred at doses ≥11 Gy; no ARS morbidity was observed for males and females at the 9 and 10 Gy doses. For the combined sexes, 30-day survival was 98, 91, and 60% at 11, 12, and 13 Gy, respectively. Log-rank analysis indicated no statistically significant differences in 30 day survival for the 9, 10, 11, and 12 Gy doses as compared with control. At 13 Gy, survival was significantly lower (*p* ≤ 0.0001) when comparing either females or the combined sexes with the corresponding control group, and male survival was trending toward significance (*p* = 0.063). Males had greater survival than females at 12 and 13 Gy with this sex difference trend in ARS survival consistent across Studies 1 and 2.

DEARE-associated deaths occurred at doses ≥9 Gy, although at 9 Gy, no females and only one male became moribund (day 153). Survival of both sexes combined at 180 days was 98, 85, 15, 2, and 0% at 9, 10, 11, 12, and 13 Gy, respectively. Males had slightly greater survival than females at 10 and 11 Gy. There was a large decrease in survival at doses ≥11 Gy for both males and females. Log-rank comparisons for 180 day survival show no significant differences in survival for the 9 and 10 Gy comparisons, however survival at 11, 12, and 13 Gy for males, females and combined sexes was significantly lower when compared with controls (*p* ≤ 0.0001). In general, survival was similar for both males and females at 180 days, whereas day 30 survival was lower for females than males, particularly in the 12 and 13 Gy dose groups.

Probit estimates for 30- and 180-day survival with 95% confidence intervals are shown in Tables 5, 6, respectively. In Study 2, animals were sacrificed starting on day 42, so only the ARS associated mortality up to day 30 from Study 2 was incorporated into the probit analysis.

**Table 5:** Thirty day probit estimate for studies 1 and 2 combined.

		**LD_30/30_**	**LD_50/30_**	**LD_70/30_**
All animals	Estimated dose (Gy)	12.85	13.40	13.98
	95% CI for dose	12.70 to 12.99	13.17 to 13.64	13.61 to 14.35
	95% CI for % mortality	0.253 to 0.351	0.414 to 0.586	0.577 to 0.804
Male	Estimated dose (Gy)	13.95	15.04	16.22
	95% CI for dose	13.02 to 14.88	13.45 to 16.63	13.84 to 18.60
	95% CI for % mortality	0.162 to 0.475	0.231 to 0.769	0.310 to 0.939
Female	Estimated dose (Gy)	12.53	12.90	13.28
	95% CI for dose	12.67 to 13.38	12.75 to 13.04	13.07 to 13.49
	95% CI for % mortality	0.233 to 0.375	0.420 to 0.580	0.594 to 0.791

CI, confidence interval.

LD_x/x_ is defined as the lethal dose to X% of the population at X days; i.e., the LD_30/30_ is the estimated radiation dose to result in 30% mortality at 30 days.

**Table 6:** One hundred and eighty day probit estimate for Study 1.

		**LD_30/180_**	**LD_50/180_**	**LD_70/180_**
All animals	Estimated dose (Gy)	10.17	10.50	10.83
	95% CI for dose	9.99 to 10.35	10.33 to 10.66	10.64 to 11.02
	95% CI for % mortality	0.205 to 0.411	0.395 to 0.605	0.594 to 0.792
Male	Estimated dose (Gy)	10.16	10.54	10.94
	95% CI for dose	9.88 to 10.44	10.29 to 10.80	10.65 to 11.22
	95% CI for % mortality	0.180 to 0.447	0.364 to 0.636	0.560 to 0.815
Female	Estimated dose (Gy)	10.20	10.44	10.69
	95% CI for dose	9.99 to 10.42	10.24 to 10.65	10.46 to 10.95
	95% CI for % mortality	0.157 to 0.484	0.332 to 0.668	0.520 to 0.841

### Body weights

**Figure 2:**
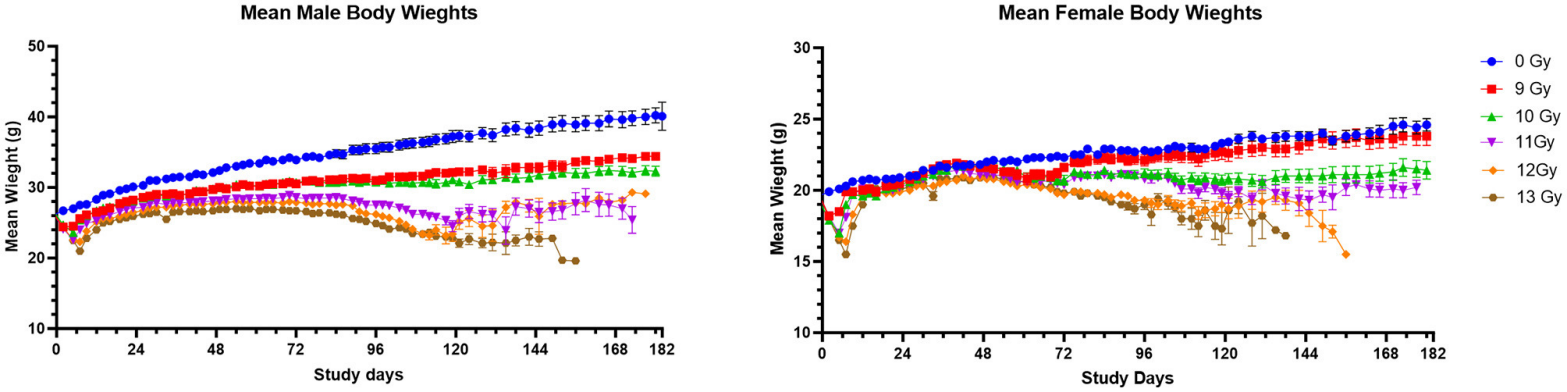
Mean body weights of PBI BM2.5 C57L/J male and female mice. Error bars represent the standard error of the mean (SEM). The number of animals for each group ranged from 24 to 126 of each sex, as shown in Table 1.

Mean body weights (BW) for male and female mice to day 180 are shown in Figure 2. In general, non-irradiated controls continuously increased in weight throughout the study with substantial weight differences between control males and females. Controls also had substantially greater body weights than the sex matched, irradiated groups. The mean BW for non-irradiated males and females reached 39 and 25 g, respectively by 180 days post-irradiation.

Body weight loss appeared in dose-dependent waves for irradiated males and females, with the first decreases generally occurring between days 2 and 14 to coincide with ARS associated morbidity. Following ARS, males irradiated at 9 and 10 Gy regained weight much slower than controls but there was no significant second period of weight loss, as there was between day 72 and 118 for males irradiated at ≥11 Gy. Females began a second dose dependent decline in weight beginning at day 42 at ≥10 Gy.

### Clinical observations

**Figure 3:**
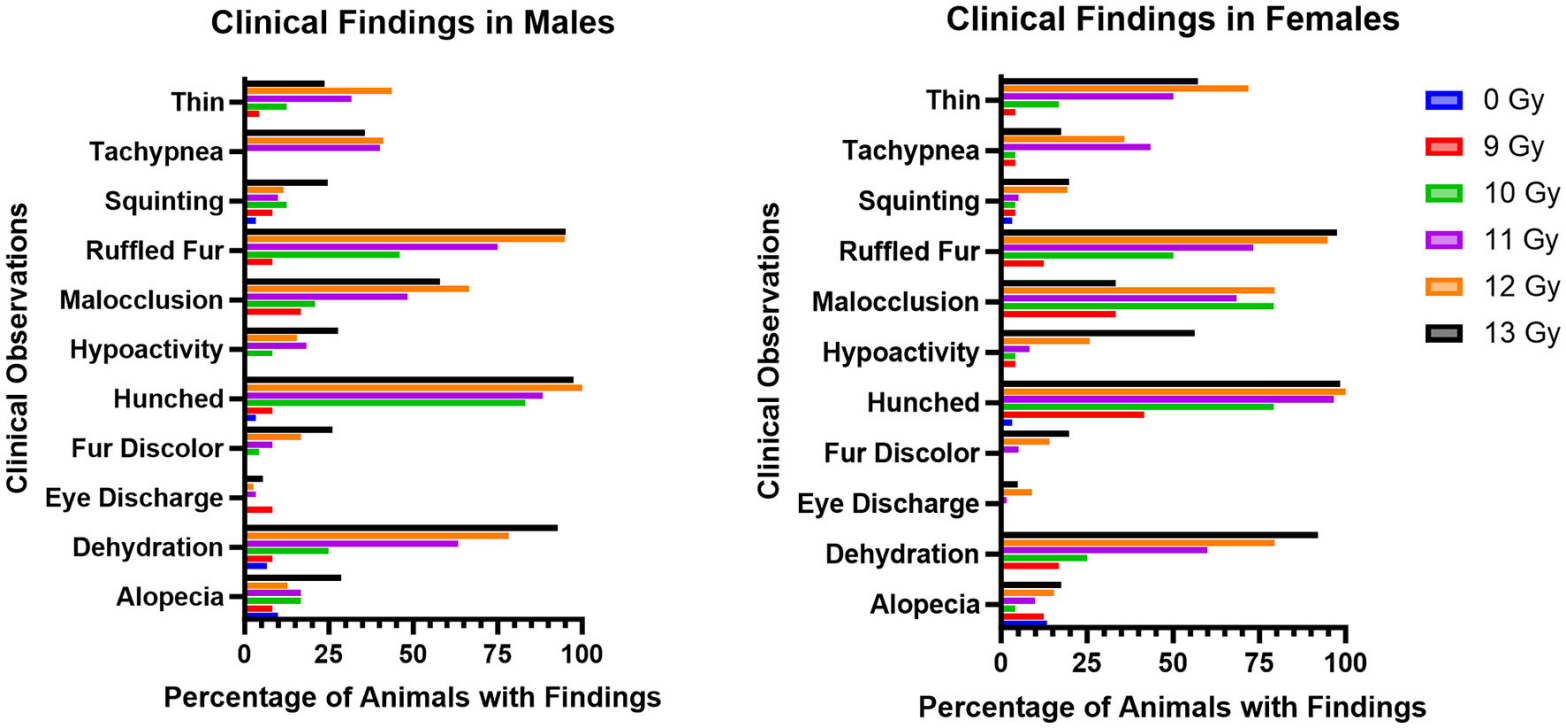
Most commonly observed clinical observations in PBI BM2.5 C57L/J male and female mice for each group, reported as the percent of animals in each group with a reported finding. The number of animals for each group ranged from 24 to 126 of each sex, as shown in Table 1.

The most common adverse clinical findings reported at any time during the study and attributed to radiation exposure in the C57L/J PBI model are shown in Figure 3. In general, the number of clinical findings increased in a dose dependent manner, with only a few sporadic findings reported in the 9 Gy dose group, and the greatest numbers of findings in the 12 and 13 Gy groups.

Ruffled fur, hunched posture, and dehydration were the most common findings, and were reported as early as 1 day following irradiation. Malocclusions were also commonly observed as early as day 49; these have also been reported as a radiation associated finding in Wistar rats (15). Malocclusions were not observed in non-irradiated controls in either animal model. Tachypnea also appeared in delayed fashion, between days 60 and 180. Other findings included fur discoloration on the unshielded portion of the body, hypoactivity, tachypnea, and a thin appearance. Corneal opacities and tumors (masses) were not observed.

### Hematology

Blood was collected from a few representative moribund animals during ARS (days 1–15) and DEARE (days 60–159) periods as unscheduled collections (Table 3). During the ARS period, the mean absolute counts for 12- and 13 Gy unscheduled moribund males and females had substantially lower values when compared with day 42 non-irradiated controls. Mean absolute values for white blood cell counts were 0.29 × 10^3^/μl for irradiated animals vs. 5.7 × 10^3^/μl for controls. Mean platelet counts were 341 × 10^3^/μl for irradiated groups vs. 1,019 × 10^3^/μl for controls, mean lymphocyte counts were 0.11 × 10^3^/μl for irradiated groups vs. 4.87 × 10^3^/μl for controls, mean monocyte counts were 0.01 × 10^3^/μl for irradiated groups vs. 0.17 × 10^3^/μl for controls, and mean neutrophil counts were 0.01 × 10^3^/μl for irradiated groups vs. 0.53 × 10^3^/μl for controls. Histopathology of bone marrow collected from these animals was markedly depleted at the time of euthanasia as shown in the heatmap of findings (Figure 6A) and representative images (Figure 6B).

Unscheduled collections from animals moribund during the DEARE period were consistent with the scheduled collections from irradiated, non-moribund animals of the same dose group.

**Figure 4:**
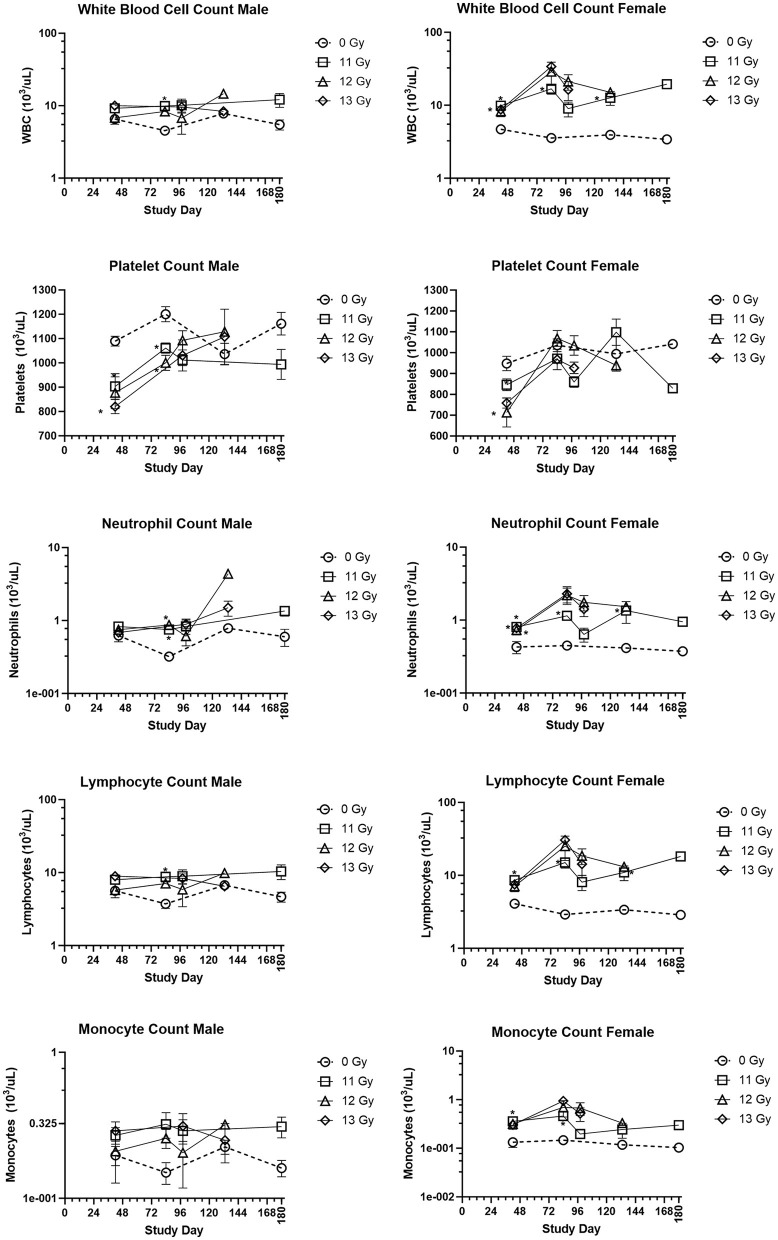
Hematology results in PBI BM2.5 C57L/J male and female mice. Group means and SEM at scheduled collection times on days 42, 84, 96, 133, and 180 for C57L/J males and females following PBI (BM2.5) X-irradiation. The numbers of animals (n) used for statistical comparisons at each timepoint ranged between 0 and 8 (Table 3). Due to the limited numbers of animals surviving to days 133 and 180 for the 12 and 13 Gy groups, statistical comparisons could not be made. The following hematology values are statistically significant (*p ≤ 0.01) as compared with non-irradiated controls: WBC for males 11 Gy at day 84, female 11 Gy at days 42, 84, and 133. Platelets: males 12 and 13 Gy day 42, 11 and 12 Gy at day 84, females 12 and 13 Gy at day 42. Lymphocytes: males 11 Gy at day 84, female 11 Gy at days 42, 84, and 133. Monocytes: female 11 Gy at days 42 and 84. Neutrophils: males 12 Gy day 84 female 11, 12, and 13 Gy at day 42, and 11 Gy at day 84 and 133.

The group means for selected hematology cell counts at scheduled collection times (days 42, 84, 98, 133, and 180) for males and females irradiated with 11, 12, and 13 Gy are presented in Figure 4. Absolute hematology values for many male and female irradiated groups and time points differed significantly from age matched non-irradiated control values, particularly at days 42 and 84. Platelet and red blood cell counts decreased for both males and females as compared with controls, mainly at day 42. White blood cell, lymphocyte, neutrophil, monocyte, basophil, and eosinophil absolute values increased for all irradiated groups as compared with controls for almost every group at each timepoint. However, because there were no control animals available for the day 98 collection, and very few 12 and 13 Gy irradiated survivors at days 133 and 180, statistical comparisons could not be made at those timepoints. Interestingly, the radiation response for absolute white blood cell and lymphocyte counts appears to be more pronounced in females.

### Clinical chemistry

**Figure 5:**
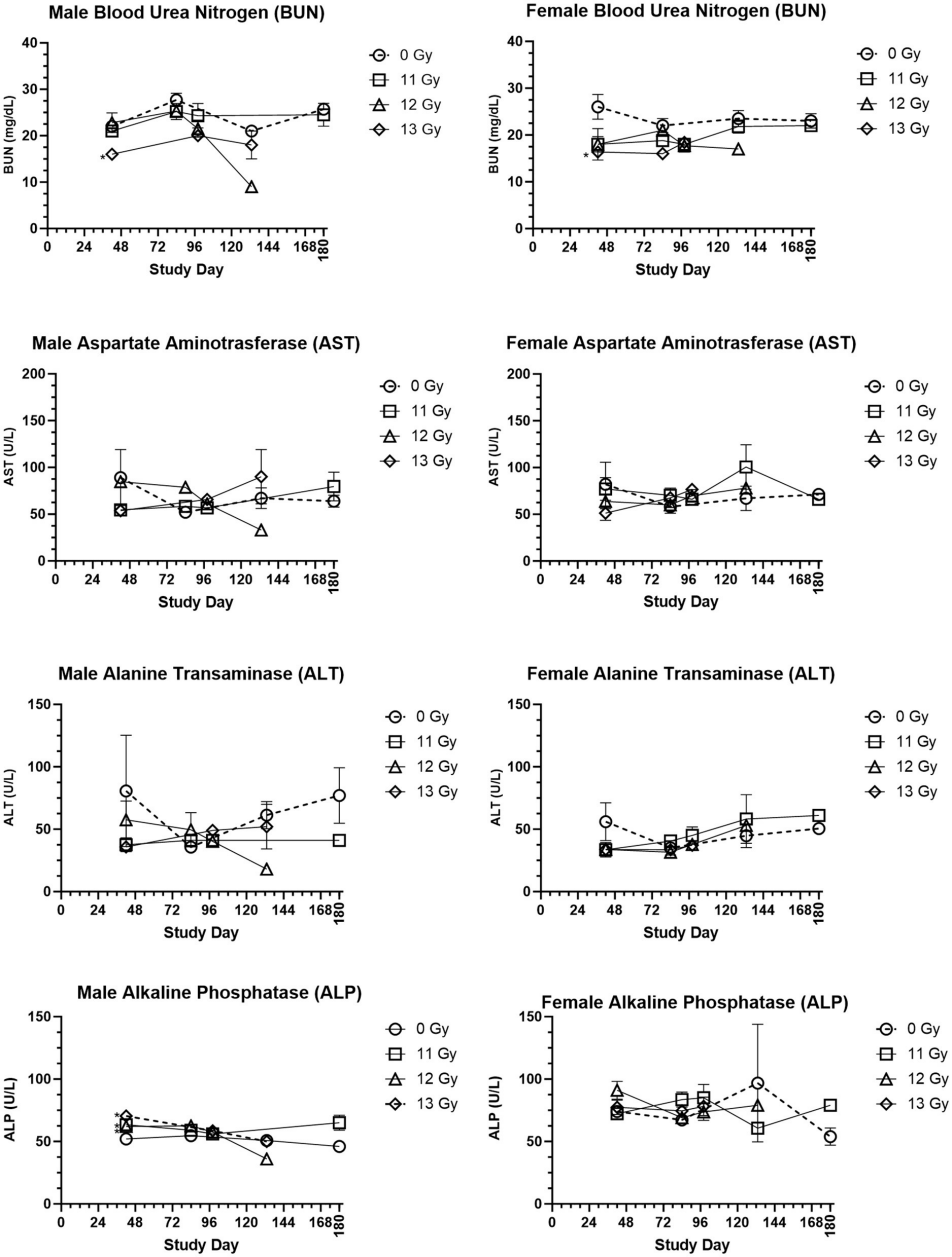
Selected clinical chemistry values of PBI BM2.5 C57L/J male and female mice. Group means and SEM at Scheduled Collection Times on days 42, 84, 96, 133, and 180 for C57L/J males and females following PBI (BM2.5) X-irradiation. The numbers of animals (n) used for statistical comparisons at each timepoint ranged between 0 and 8 (Table 3). Due to the limited numbers of animals surviving to days 133 and 180 for the 12 and 13 Gy groups, statistical comparisons could not be made. Statistically significant differences (*p ≤ 0.01) as compared with non-irradiated controls were detected for the following parameters and timepoints: BUN for males and females at 13 Gy day 42, ALP for males at 11, 12, and 13 Gy at Day 42.

Clinical chemistry parameters are presented in Figure 5. Increases in blood urea nitrogen (BUN) and creatinine are generally indicative of kidney injury. In the C57L/J model, values were generally lower in irradiated male and females as compared with age matched, non-irradiated controls, yet these differences were not statistically significant for most groups and timepoints. Only at day 42 was there a significantly reduced BUN value for 13 Gy irradiated males and females. BUN values were also similar to controls for males and females euthanized for cause during the ARS response period, but many of those euthanized due to DEARE showed values lower than similarly aged controls for both males and females. Creatinine values were below the limit of detection across nearly all scheduled and unscheduled collections.

Changes in alanine transaminase (ALT), aspartate aminotransferase (AST), and alkaline phosphatase (ALP) often indicate liver injury. In this study, ALT and AST levels for male and female irradiated groups at scheduled collections and in the representative samples from animals euthanized for cause during the ARS and DEARE periods were similar to controls. ALP values did reveal some statistically significant increases for males irradiated at 11, 12, and 13 Gy at day 42, but there were no other statistically significant differences for males at any other timepoints, or for females at any time. This is consistent with few findings of liver injury in histopathology samples.

Blood glucose was slightly reduced in irradiated males and females, this was most pronounced in males at day 133 and 168. However, the only statistically significant reduction was for 13 Gy males at day 98. In males and females euthanized for cause, glucose levels were lower than age matched controls.

Cholesterol in irradiated males was generally reduced as compared with controls, and this reduction was statistically significant for 11 and 12 Gy groups at day 84. In contrast, irradiated females had slightly higher cholesterol values than controls, although this was not significantly different.

Albumin/globulin ratios were generally similar between age matched controls and irradiated males and females at most time points. The only exception was at 19 weeks, when 13 Gy males and 11 Gy females were significantly reduced as compared with controls. Among animals euthanized for cause, the albumin/globulin ratio for males was generally reduced as compared with controls, however for females the values were much more variable and some animals displayed values much greater or lower than controls.

Triglycerides showed a general trend of being reduced in irradiated male and female groups, however none of these comparisons were statistically significant.

Other parameters, including sodium, potassium, total protein, globulin, albumin, chloride and calcium were also examined. There was no clear trend to these data, making it difficult to determine if or how these changes are a result of radiation injury.

### Histopathology

**Figure 6:**
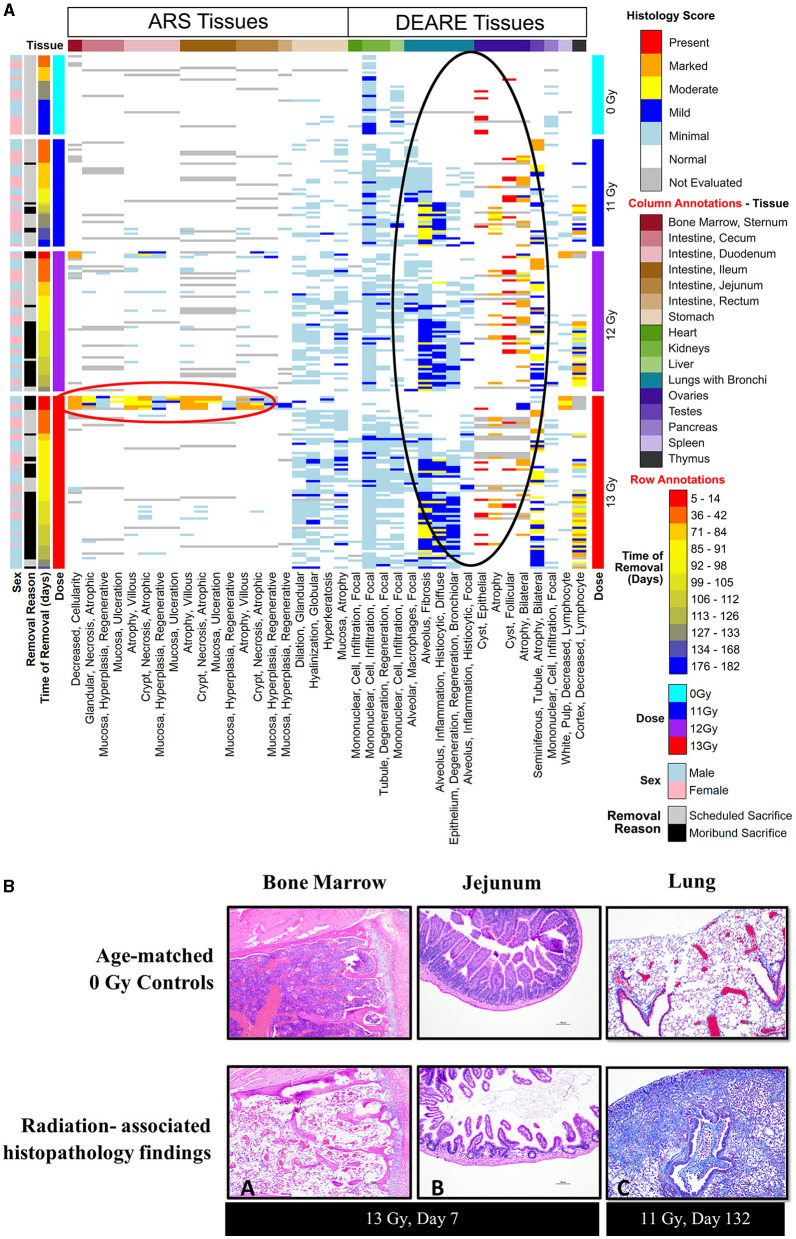
**(A, B)** Histopathology heat map **(A)** and histopathology images **(B)** from of PBI BM2.5 C57L/J male and female mice. **(A)** Histopathology tissue samples were collected at pre-determined intervals from a minimum of 3 mice/sex for non-irradiated controls 0 Gy, 11, 12, and 13 Gy irradiated animals (left axis, light blue, dark blue, purple, and red bars, respectively) at 42, 84, 98, 133, and 182 days post-irradiation, and from moribund animals when possible. Findings of reduced bone marrow cellularity and GI tissue injury are most prevalent in the 1st week following 13 Gy PBI (red circle). In the lung, testis and ovaries, findings of lung inflammation and fibrosis and gonadal atrophy are prevalent in irradiated animals generally after day 84/week 12 (black circle). **(B)** Histopathology images: findings of reduced bone marrow cellularity (A) and villi/crypt degeneration in jejunum (B) are observed at 7 days post −13 Gy PBI irradiation (H&E stain). (C) Trichrome stain of DEARE-associated lung fibrosis at day 132. Mild to moderate alveolar fibrosis is a common DEARE finding amongst animals irradiated at ≥11 Gy PBI.

Histopathology results are shown in Figure 6, along with representative H&E stained images of bone marrow (sternum) and GI tissue (jejunum) during ARS, and Masson's trichrome stained lung sections during DEARE. Histopathological findings were generally consistent when comparing males and females across all three radiation dose groups, and the numbers and severity of DEARE findings generally increased over time in a dose-dependent fashion.

In animals euthanized due to ARS prior to day 30, the primary findings were intestinal necrosis and hematopoietic atrophy associated with the sequalae of GI- and H-ARS.

For animals euthanized during the DEARE period, the predominant causes of death were pulmonary inflammation and fibrosis associated with DEARE. Among the irradiated groups, the types of findings reported were consistent over time, and most prevalent in lung and kidney, with stomach and liver having relatively fewer findings. Findings of atrophy in ovaries and testicles were also common in all the irradiated, necropsied animals. There were a small number of sporadic findings scored as minimal in the bone marrow, GI tissues, heart, pancreas, thymus, spleen and bladder.

In scheduled collections at 42 days, kidney findings were primarily of minimal tubule degeneration/regeneration seen in 18 of 30 irradiated animals, and minimal monocyte infiltration which was similarly prevalent in both controls and irradiated animals. Tubule degeneration was more abundant in females. There were a few lung findings of minimal alveolar fibrosis and minimal alveolar inflammation (six of 30 irradiated animals), minimal focal alveolar macrophages was the most common finding, seen in 20 of 30 irradiated animals. In the spleen, findings were of increased hematopoiesis in 21 of 30 irradiated animals; in males this was scored as minimal, in females the majority were scored as mild, indicating a slightly greater response in females. There were numerous findings in the stomach of 20 of 30 irradiated animals, including minimal glandular dilation, minimal neutrophilic focal infiltration, minimal hyperkeratosis, minimal mucosal atrophy, and minimal globular hyalinization.

At day 84, kidney tubule degeneration was noted only in 11 of 18 irradiated animals, was again more prevalent in females, and almost entirely scored as minimal. Lung findings of minimal alveolar fibrosis increased in number at day 84 affecting 15 of 18 irradiated animals with numbers distributed relatively equally across all male and female groups; alveolar inflammation and focal alveolar macrophages also were noted but less prevalent in each group. The number of stomach findings was reduced, present in only seven of 18 irradiated animals. In the liver, minimal focal mononuclear cell infiltration was present in all nine irradiated females and only three of nine irradiated males.

At day 98, kidney findings of tubule degeneration increased in number, and was seen in 23 of 28 irradiated animals. The number of lung findings of minimal and mild alveolar fibrosis increased, affecting all 28 of the irradiated animals examined. Minimal or mild focal alveolar inflammation was present in the majority of animals (18 of 28), and focal alveolar macrophages were also noted in nearly every irradiated animal (22 of 28). Stomach findings were reported in 24 of 28 irradiated animals at day 98. Liver findings were seen in the 12 and 13 Gy groups in 17 of 22 animals.

At day 133, only non-irradiated controls, four irradiated (1 at 12 Gy, 3 at 13 Gy) males and seven irradiated (6 at 11 Gy, 1 at 12 Gy) females remained. Kidney findings in irradiated animals were primarily of minimal tubule degeneration/regeneration in all males and nearly all females. In lung, increasingly severe alveolar fibrosis was present in all irradiated males and females. Other lung findings such as alveolar inflammation and epithelium degeneration/regeneration continued to be reported. Liver findings continued to show minimal focal mononuclear cell infiltrations in controls as well in six of 11 irradiated animals.

At the 180-day scheduled collection, only controls and three 11 Gy animals remained, and findings were consistent with the previous day 133 collection.

### Magnetic resonance imaging (MRI)

**Figure 7:**
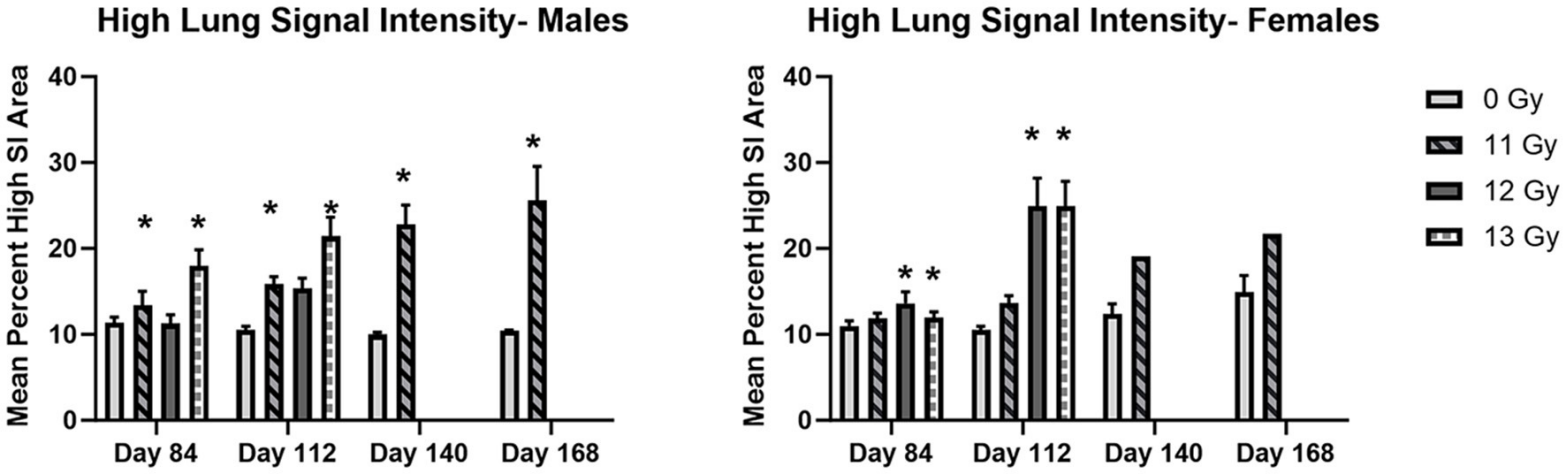
MR image analysis of PBI BM2.5 C57L/J male and female mice irradiated at ≥11 Gy. Areas of high signal intensity indicating possible congestion, inflammation and fibrosis associated with L-DEARE are significantly larger (*) in irradiated animals as compared to controls for both males and females. The number of animals for each group comparison ranges from 0 to 8 (Table 2), no 12 or 13 Gy animals in the MRI cohort survived to day 140 and 168. * indicates statistically significant difference (*p* ≤ 0.05) in high lung signal intensity area as compared to the controls (0 Gy) by 2-way ANOVA and Tukey comparison of means.

MRI results are shown in Figure 7. Analysis of T2 images showed a general trend of increased area of high signal intensity (SI) which may indicate congestion, inflammation, or fibrosis in irradiated animals as compared with non-irradiated controls.

Statistically significant increases in high SI area as compared with controls were seen at days 84 and 112 in 11 and 12 Gy irradiated males and 12 and 13 Gy irradiated females. At days 140 and 168, 11 Gy males have a statistically significant increase in high SI area, and although 11 Gy females showed a similar increase, there were too few animals remaining for statistical significance.

## Discussion

The objective of the current study was to further characterize the C57L/J mouse model of DEARE that can be used for the development of MCMs to be submitted for approval under the FDA Animal Rule regulatory pathway. To be acceptable under the Animal Rule, it is paramount that the animal model be well-characterized and reflective of human clinical condition because efficacy of the MCM will be assessed in animals, not humans, due to ethical concerns. To that end, the natural history and dose response relationship of the PBI BM2.5 C57L/J model following X-irradiation was characterized as described herein.

The acute radiation response in the PBI BM2.5 C57L/J mouse model shows many characteristics common to TBI and PBI models of H- and GI-ARS in other strains of mice, in rats, and non-human primates. Leukocytopenia just days after irradiation is a common finding in TBI C57BL6 mice (16) and rhesus macaques (17), PBI C57BL6 (18), and C57 L/J (10) mice, Wistar rats (15) and rhesus macaques (19, 20). In the PBI L/J model presented here, hematopoietic atrophy and gastrointestinal necrosis are clearly observed in the hematology values and histopathology tissues collected from ARS moribund mice (Figure 6B). These are well-documented, deterministic events common to PBI resulting in H- and GI-ARS for both the C57BL6 (21), and C57 L/J (10).

### ARS survival

C57L/J mice are susceptible to ARS associated morbidity in a dose-dependent fashion at doses ≥11 Gy for males and ≥12 Gy for females. In this model, females are more susceptible to ARS mortality than males at the same doses (Table 4). This sex difference is reflected in the probit estimates of radiation dose for 30, 50, and 70 percent lethality at day 30, wherein the probit estimates for radiation dose are substantially higher for males than females (Table 5). Although doses at ≥11 Gy can result in H- and GI-ARS associated morbidity, 30-day morbidity is relatively low as compared with 180-day morbidity for both males and females.

### One hundred and eighty day DEARE survival

For animals surviving past the H- and GI-ARS stage (day 30), pathology exams indicated the cause of mortality was likely L-DEARE, which occurred between days 60 and 159 (Figure 1). This period of mortality coincides with the appearance of lung fibrosis and inflammation in male and female histopathology samples at doses of ≥11 Gy (Figure 6). Clinical indications of inflammation and/or fibrosis can also be quantified in MR images with both males and females having significant greater area of high contrast/density/signal intensity lung tissue (Figure 7). L-DEARE morbidity has also been reported in similar C57L/J PBI models beginning ~10 weeks after exposure at 9.5 Gy and higher (10), supporting the evidence presented here of a deterministic radiation response of the C57L/J mouse lungs at the doses applied. Interestingly, there were no sex differences in survival at day 180, and probit estimates for the LD30,−50, or−70 are very similar for males and females (Table 6).

In addition to L-DEARE, hematology values indicate persistent dysregulation of the hematopoietic compartment well past the typical H-ARS 30-day period. Although persistent hematological dysregulation appears in ARS survivors, the changes that manifest may differ for TBI and PBI models in mice. For instance, in TBI C57BL6 mice circulating blood cell values are persistently reduced during the DEARE period for neutrophils, lymphocytes, red blood cells and platelets (4). In contrast, the PBI Wistar rat (15) and the L/J mouse model presented show increases in white blood cells, lymphocytes and neutrophils. It has been proposed that the persistent changes seen in the hematological compartment as delayed effect of irradiation may be the result of residual bone marrow damage (RBMD), as first described in the TBI C57BL6 (4, 22) and more recently, other models (2). However, it is not yet clear if the differences observed in DEARE hematology for the TBI C57BL6 and PBI C57 L/J models are due to intrinsic strain differences in the radiation response, differences between TBI vs. PBI models, such as the radiation dose applied, or volume of bone marrow irradiated, and/or if RBMD differs by strain, species, or the irradiation model used.

It is well-known that the hematopoietic compartment and bone marrow are very sensitive to radiation injury. In this PBI model, we observed reduced bone marrow cellularity acutely in ARS moribund animals after ≥12 Gy exposures, and the temporary decline in marrow cellularity rebounded in histopathology samples collected at 42 days after irradiation. Despite the apparent regeneration, there were persistent and significant changes in the absolute counts of cell types that were depleted during ARS (i.e., white blood cells, platelets, lymphocytes, monocyte, and neutrophil counts). At scheduled and unscheduled collections following the ARS period, white blood cells, lymphocytes, neutrophils, and monocytes were all increased in both sexes ≥11 Gy irradiated groups as compared with non-irradiated controls across nearly all time points examined while platelet counts were slightly, but significantly decreased, in irradiated animals. Whether these changes are because of persistent damage present in the repopulated bone marrow, and/or crosstalk between other irradiated and persistently damaged tissues remains unclear. However, there was no apparent leukemia, anemia, or leukocytopenia at scheduled collections, making it difficult to determine how or if persistent hematological dysregulation contributes to the overall DEARE morbidity in this model.

Chronic kidney disease following irradiation develops clinically, and both PBI rat (23) and PBI NHP (20) models develop K-DEARE either concurrent to, or following, L-DEARE. In the C57L/J mouse model described herein, there is a lack of evidence for the development of K-DEARE, and this is consistent with the similar C57L/J model developed by Gibbs et al. (10). Histopathology findings of were primarily of minimal tubule degeneration. Although the number of findings of kidney injury was increased at the highest radiation dose, tissue scores indicating the severity of injury were rated as minimal for nearly all animals regardless of radiation dose. Furthermore, serum BUN values, a marker that typically increases with kidney damage, were either similar to or reduced in irradiated animals as compared with controls (Figure 5). Another marker of kidney function, serum creatinine (not shown) was below the lower limit of quantitation in all animals including controls. Although findings of K-DEARE were not present, the very high mortality that occurred due to L-DEARE greatly reduced the numbers of animals available to potentially develop K-DEARE later. Thus, there is insufficient evidence to rule out the possibility of K-DEARE developing in this strain, possibly at a lower radiation dose or over a longer time period than examined here.

These findings support utility of the model for L-DEARE countermeasures testing following ARS recovery. The major benefits to the use of this model are the relatively low ARS mortality at doses needed to result in the consistent and deterministic lung response of pulmonary inflammation/pneumonitis and fibrosis, with very little supportive care (moistened food and hydrogel hydration supplement) provided. H-ARS mortality may be further reduced through the use of approved H-ARS MCMs, and the use of such supportive care could be provided at the discretion of the investigator. In this way, efficacy of L-DEARE MCMs can be tested in the context of the multi-organ injury that results from PBI, either with or without the provision of supportive care in the form of an approved ARS countermeasure.

## Data availability statement

The datasets presented in this article are not readily available because, proprietary data owned by NIAID, only available with NIAID permission. Requests to access the datasets should be directed to David Cassatt, NIAID COR, cassattd@niaid.nih.gov.

## Ethics statement

The animal study was approved by SRI International IACUC Committee. The study was conducted in accordance with the local legislation and institutional requirements.

## Author contributions

TB: Writing – original draft. JB: Writing – review & editing. JM: Writing – review & editing. ER: Writing – review & editing. HJ: Formal analysis, Writing – review & editing. DN: Writing – review & editing. SK: Writing – review & editing. DB: Writing – review & editing. PC: Writing – review & editing.

## References

[1] Williams JP McBride WH. After the bomb drops: a new look at radiation-induced multiple organ dysfunction syndrome (Mods). Int J Radiat Biol. (2011) 87:851–68. 10.3109/09553002.2011.560996 21417595 PMC3314299

[2] Wu T Orschell CM. The delayed effects of acute radiation exposure (DEARE): characteristics, mechanisms, animal models, and promising medical countermeasures. Int J Radiat Biol. (2023) 99:1066–79. 10.1080/09553002.2023.2187479 36862990 PMC10330482

[3] FDA. Guidance for Industry: Product Development Under the Animal Rule. Approval of New Drugs When Human Efficacy Studies Are Not Ethical or Feasible. 21 Cfr 314.600-650 for Drugs; 21 Cfr 601.90-95 for Biologics. (2002). Available online at: https://www.ecfr.gov/current/title-21/part-314/subpart-I (accessed November 20, 2023).

[4] Chua HL Plett PA Fisher A Sampson CH Vemula S Feng H. Lifelong residual bone marrow damage in murine survivors of the hematopoietic acute radiation syndrome (H-Ars): a compilation of studies comprising the Indiana University experience. Health Phys. (2019) 116:546–57. 10.1097/HP.0000000000000950 30789496 PMC6388630

[5] Singh VK Newman VL Seed TM. Colony-stimulating factors for the treatment of the hematopoietic component of the acute radiation syndrome (H-Ars): a review. Cytokine. (2015) 71:22–37. 10.1016/j.cyto.2014.08.003 25215458

[6] Bunin DI Javitz HS Gahagen J Bakke J Lane JH Andrews DA. Survival and hematologic benefits of romiplostim after acute radiation exposure supported FDA approval under the animal rule. Int J Radiat Oncol Biol Phys. (2023) 117:705–17. 10.1016/j.ijrobp.2023.05.008 37224926

[7] Chua HL Plett PA Sampson CH Katz BP Carnathan GW MacVittie TJ. Survival efficacy of the pegylated G-Csfs Maxy-G34 and neulasta in a mouse model of lethal H-Ars, and residual bone marrow damage in treated survivors. Health Phys. (2014) 106:21–38. 10.1097/HP.0b013e3182a4df10 24276547 PMC3843155

[8] Williams JP Jackson IL Shah JR Czarniecki CW Maidment BW DiCarlo AL. Animal models and medical countermeasures development for radiation-induced lung damage: report from an Niaid Workshop. Radiat Res. (2012) 177:e0025–39. 10.1667/RROL04.1 22468702 PMC8365776

[9] Satyamitra MM Cassatt DR Marzella L. A trans-agency workshop on the pathophysiology of radiation-induced lung injury. Radiat Res. (2022) 197:408–14. 10.1667/RADE-21-00153.1 34714907 PMC9063325

[10] Gibbs A Gupta P Mali B Poirier Y Gopalakrishnan M Newman D. A C57l/J mouse model of the delayed effects of acute radiation exposure in the context of evolving multi-organ dysfunction and failure after total-body irradiation with 25% bone marrow sparing. Radiat Res. (2023) 199:319–35. 10.1667/RADE-22-00178.1 36857032 PMC10289057

[11] Rabender CS Mezzaroma E Yakovlev VA Mauro AG Bonaventura A Abbate A. Mitigation of radiation-induced lung and heart injuries in mice by oral sepiapterin after irradiation. Radiat Res. (2021) 195:463–73. 10.1667/RADE-20-00249.1 33822229 PMC8162938

[12] Jackson IL Xu P Hadley C Katz BP McGurk R Down JD. Preclinical rodent model of radiation-induced lung injury for medical countermeasure screening in accordance with the FDA animal rule. Health Phys. (2012) 103:463–73. 10.1097/HP.0b013e31826386ef 22929472 PMC3604892

[13] Jackson IL Vujaskovic Z Down JD. Revisiting strain-related differences in radiation sensitivity of the mouse lung: recognizing and avoiding the confounding effects of pleural effusions. Radiat Res. (2010) 173:10–20. 10.1667/RR1911.1 20041755 PMC2818983

[14] Jackson IL Vujaskovic Z Down JD. A further comparison of pathologies after thoracic irradiation among different mouse strains: finding the best preclinical model for evaluating therapies directed against radiation-induced lung damage. Radiat Res. (2011) 175:510–18. 10.1667/RR2421.1 21338245 PMC3110676

[15] Beach T Bakke J Riccio E Javitz HS Nishita D Kapur S. The progression of radiation injury in a Wistar Rat Model of partial body irradiation with approximately 5% bone marrow shielding. Int J Radiat Biol. (2023) 99:1080–95. 10.1080/09553002.2023.2188937 36930794

[16] Plett PA Sampson CH Chua HL Joshi M Booth C Gough A. Establishing a murine model of the hematopoietic syndrome of the acute radiation syndrome. Health Phys. (2012) 103:343–55. 10.1097/HP.0b013e3182667309 22929467 PMC3743168

[17] Farese AM Hankey KG Cohen MV MacVittie TJ. Lymphoid and myeloid recovery in rhesus macaques following total body X-irradiation. Health Phys. (2015) 109:414. 10.1097/HP.0000000000000348 26425902 PMC4593069

[18] Kumar VP Wuddie K Tsioplaya A Weaver A Holmes-Hampton GP Ghosh SP. Development of a multi-organ radiation injury model with precise dosimetry with focus on Gi-Ars. Radiat Res. (2024) 201:19–34. 10.1667/RADE-23-00068.1 38014611

[19] Farese AM Bennett AW Gibbs AM Hankey KG Prado K Jackson W 3rd. Efficacy of neulasta or neupogen on H-Ars and Gi-Ars mortality and hematopoietic recovery in nonhuman primates after 10-Gy irradiation with 25% bone marrow sparing. Health Phys. (2019) 116:339–53. 10.1097/HP.0000000000000878 30281533 PMC6349470

[20] MacVittie TJ Farese AM Parker GA Bennett AW Jackson WE 3rd. Acute radiation-induced lung injury in the non-human primate: a review and comparison of mortality and co-morbidities using models of partial-body irradiation with marginal bone marrow sparing and whole thorax lung irradiation. Health Phys. (2020) 119:559–87. 10.1097/HP.0000000000001346 33009295 PMC9440605

[21] Booth C Tudor G Tudor J Katz BP MacVittie TJ. Acute gastrointestinal syndrome in high-dose irradiated mice. Health Phys. (2012) 103:383–99. 10.1097/HP.0b013e318266ee13 23091876 PMC3530834

[22] Wu T Plett PA Chua HL Jacobsen M Sandusky GE MacVittie TJ. Immune reconstitution and thymic involution in the acute and delayed hematopoietic radiation syndromes. Health Phys. (2020) 119:647–58. 10.1097/HP.0000000000001352 32947490 PMC7541734

[23] Gasperetti T Miller T Gao F Narayanan J Jacobs ER Szabo A. Polypharmacy to mitigate acute and delayed radiation syndromes. Front Pharmacol. (2021) 12:634477. 10.3389/fphar.2021.634477 34079456 PMC8165380

